# Impact of Sarcopenia on Treatment Outcomes and Toxicity in Locally Advanced Rectal Cancer

**DOI:** 10.3390/medicina60101606

**Published:** 2024-10-01

**Authors:** Sebastian Curcean, Alexandra Gherman, Alexandru Tipcu, Zsolt Fekete, Alina-Simona Muntean, Andra Curcean, Rares Craciun, Stefan Stanciu, Alexandru Irimie

**Affiliations:** 1 Department of Radiation Oncology, Iuliu Hatieganu University of Medicine and Pharmacy, 8 Victor Babes Street, 400012 Cluj-Napoca, Romania; 2 Department of Radiation Oncology, “Prof. Dr. Ion Chiricuta” Oncology Institute, 34–36 Republicii Street, 400015 Cluj-Napoca, Romania; 3 Department of Medical Oncology, Iuliu Hatieganu University of Medicine and Pharmacy, 8 Victor Babes Street, 400012 Cluj-Napoca, Romania; 4 Department of Medical Oncology, “Prof. Dr. Ion Chiricuta” Oncology Institute, 34–36 Republicii Street, 400015 Cluj-Napoca, Romania; 5 Faculty of Medicine, Iuliu Hatieganu University of Medicine and Pharmacy, 8 Victor Babes Street, 400012 Cluj-Napoca, Romania; 6 Department of Imaging, Affidea Center, 15c Ciresilor Street, 400487 Cluj-Napoca, Romania; 7 Department of Internal Medicine, Iuliu Hatieganu University of Medicine and Pharmacy, 8 Victor Babes Street, 400012 Cluj-Napoca, Romania; 8 Gastroenterology Department, “Prof. Dr. O. Fodor” Regional Institute of Gastroenterology and Hepatology, 400162 Cluj-Napoca, Romania; 9 Department of Oncological Surgery and Gynecological Oncology, Iuliu Hatieganu University of Medicine and Pharmacy, 8 Victor Babes Street, 400012 Cluj-Napoca, Romania; 10 Department of Oncological Surgery, “Prof. Dr. Ion Chiricuta” Oncology Institute, 34–36 Republicii Street, 400015 Cluj-Napoca, Romania

**Keywords:** sarcopenia, locally advanced rectal cancer, pathological complete response, clinical complete response, toxicity

## Abstract

*Background and Objectives*: Sarcopenia, a condition characterized by muscle mass loss, is prevalent in up to 68% of rectal cancer patients and has been described as a negative prognostic factor, impacting overall survival and tumor response. While there are extensive data on rectal cancer globally, only a handful of studies have evaluated the role of sarcopenia in locally advanced rectal cancer (LARC). Our study aimed to investigate the relationship between sarcopenia, overall response rate, and toxicity in patients who underwent total neoadjuvant treatment (TNT) for LARC. *Materials and Methods*: We performed a retrospective study of patients with rectal cancer treated with TNT and surgery with curative intent between 2021 and 2023 at Prof. Dr. Ion Chiricuta Institute of Oncology, Cluj-Napoca. Sarcopenia was assessed on MRI images by measuring the psoas muscle area (PMA) at the level of the L4 vertebra before and after neoadjuvant therapy. The primary endpoints were the overall complete response rate (oCR) and acute toxicity. *Results*: This study included 50 patients with LARC. The oCR rate was 18% and was significantly associated with post-treatment sarcopenia (OR 0.08, *p* = 0.043). Patients who did not achieve a clinical or pathologic complete response had, on average, an 8% muscle loss during neoadjuvant therapy (*p* = 0.022). Cystitis and thrombocytopenia were significantly associated with post-treatment sarcopenia (*p* = 0.05 and *p* = 0.049). *Conclusions*: Sarcopenia and loss of psoas muscle during neoadjuvant therapy were negatively associated with tumor response in locally advanced rectal cancer. Thrombocytopenia and cystitis are more frequent in sarcopenic than non-sarcopenic patients undergoing neoadjuvant chemoradiation for rectal cancer.

## 1. Introduction

Despite consistent therapeutic advancements in the past three decades, locally advanced rectal cancer (LARC) remains a challenge. Local recurrences amount to 5%, with distant metastasis rates being fourfold, translating to overall survival (OS) rates of 80% at 5 years [1,2]. Total neoadjuvant therapy (TNT) is an attempt to mitigate this outcome by delivering chemotherapy pre-operatively, either before or after radiotherapy. This novel treatment strategy has the potential to improve disease-free survival rates, secure adherence to chemotherapy, and increase the rates of organ preservation among a substantial proportion of patients achieving a complete clinical response (cCR) [3,4].

Sarcopenia is characterized by a progressive loss of skeletal muscle mass and function, causing functional decline and increased mortality [5]. Sarcopenia can be classified as primary sarcopenia when it is related to old age and secondary sarcopenia when it is disease-associated [6]. EWGSOP2 guidelines recommend that primary sarcopenia is diagnosed by a three-step approach as follows: Low muscle strength indicates probable sarcopenia; diagnosis is confirmed by assessing muscle quantity or quality; and finally, severity is graded by physical performance [7]. Secondary sarcopenia has predominantly focused on loss of muscle mass [6]. Muscle strength has been evaluated by grip strength or chair stand test [7,8]. Muscle quality and/or quantity is assessed by dual-energy X-ray absorptiometry (DXA) or lumbar muscle cross-sectional area by CT or MRI [7,9]. Lastly, physical performance is tested using several approaches, like gait speed or short physical performance battery [7]. Management of sarcopenia is complex, with physical exercise being the backbone of sarcopenia treatment [10]. The role of nutrition is less clear, and pharmacological compounds are currently under research, such as anabolic catabolic transforming agents [11]. Evidence indicates that the metabolic disturbances caused by tumors may drive the molecular dysregulation that leads to muscle atrophy [12]. Timely detection of sarcopenia is crucial for enhancing patient care and the overall prognosis.

In rectal cancer, sarcopenia can be present in up to 68% of patients and leads to a significant decrease in overall survival [13]. Although there are extensive data on sarcopenia and colorectal cancer, only limited evidence exists on the impact of sarcopenia on locally advanced rectal cancer. To our knowledge, there is only one prospective study on an Australian cohort describing the negative association between sarcopenia and treatment response [14] and two retrospective studies on Turkish and Australian cohorts, the former showing a sevenfold increase in pathological complete response in non-sarcopenic patients compared to sarcopenic patients [15], while the latter showed no significant difference in tumor regression grade between the groups [16].

Our study aimed to investigate the impact of MRI-defined sarcopenia on pathological and clinical complete response rates following total neoadjuvant treatment and subsequent toxicities in patients with LARC. The hypothesis was that sarcopenic patients would have lower rates of complete responses.

## 2. Materials and Methods

### 2.1. Patient Selection

Fifty consecutive patients over the age of 18 diagnosed with locally advanced rectal cancer (i.e., stage II and III as per AJCC 8th edition and patients with stage I who declined surgery) and treated in our institution between 2021 and 2023 were selected from the institutional database. All patients received long-course chemoradiation (nCRT) either preceded by induction chemotherapy (INCT) or succeeded by consolidation chemotherapy (CNCT) or both. Long-course chemoradiation consisted of 50 Gy in 25 fractions administered with the IMRT simultaneous integrated boost (SIB) technique over the course of five weeks with concurrent Capecitabine 825 mg/m^2^ BID or weekly Fu-Fol (folinic acid 30 mg/m^2^ and 5 FU 600 mg/m^2^). Induction or consolidation chemotherapy was proposed for a total of either 8 cycles of FOLFOX4 over 16 weeks or 6 cycles of CAPOX over the course of 18 weeks. However, patients who did not complete the full protocol were accepted. All cases were discussed in a multidisciplinary team that meets weekly. The first reassessment was performed at 8 weeks with CEA and MRI scan. Surgery was offered to all patients who did not achieve a complete clinical response as assessed by MRI tumor regression grade (mrTRG1), proctoscopy, and CEA at 8 weeks from completion of nCRT. Patients with a near-complete response (mrTRG2) were reevaluated at 4 weeks from the prior MRI [17,18]. Patients with stage I disease who refused upfront surgery were offered TNT. Only cases with available staging and reassessment MRI scans were included.

### 2.2. Sarcopenia Measurement

**Figure 1:**
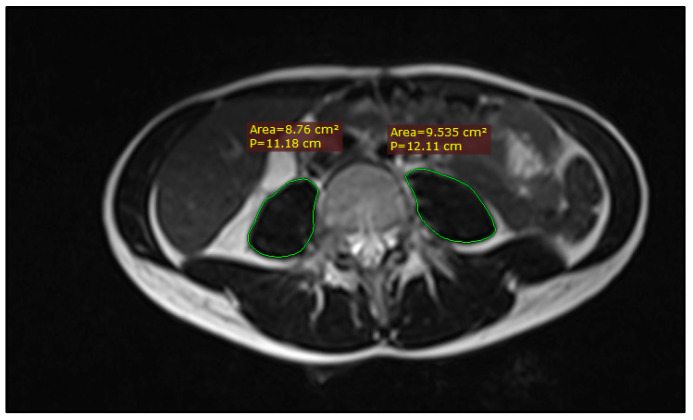
Example of PMA measuring on an axial slice of a T2-weighted MRI image at the level of L4.

Sarcopenia was assessed on two MRI imaging sets for each patient: pre-treatment staging scans and post-treatment response assessment scans at 8 weeks from completion of chemoradiation. The psoas muscle was delineated bilaterally on T2-weighted axial images using the closed polygon tool in RadiAnt DICOM viewer v.2024.1 at the mid-vertebral L4 level, as shown in Figure 1. The resulting psoas muscle area (PMA) was used to ascertain sarcopenia based on the Sarcopenia t-score calculator, developed by Derstine et al. [7,9,19]. As the calculator was developed for CT-based measurements, 10 random cases were selected to compare L4 PMA on pre-treatment simulation CT scans and L4 PMA on pre-treatment MRI scans, which showed no significant differences.

### 2.3. Statistical Analysis

Distribution analysis was performed using Kolmogorov–Smirnov and Shapiro–Wilk tests, along with the distribution charts. Assessment for independence was performed by the means of Chi-square/exact Fisher testing (applying Yates’ correction for continuity when needed). Dependent groups were compared using a paired sample t-test. Independent groups were compared using independent samples *t*-test or Mann–Whitney U test. Linear relationships were tested using Spearman’s correlations.

All testing was performed using IBM SPSS Statistics v.26.0.0.

### 2.4. Outcome Evaluation

Primary endpoints were cCR and pCR, reunited under overall complete response (oCR) and overall toxicity after TNT. Overall complete response represents the sum of clinical and pathological complete responses. Clinical complete response was defined as no palpable tumor on digital rectal examination, no visible tumor and a pale scar on proctoscopy, mrTRG 1 or 2 on reassessment MRI, with no evidence of pathologic lymph nodes or EMVI [20,21]. The pathological complete response was defined as the absence of viable cells on the surgical specimen (ypT0N0), modified Ryan grade 0. Acute toxicity was documented based on CTCAE 5.0.; the worst grade of hematological, nervous system, digestive, and radiation-induced proctitis or cystitis was recorded over the course of the whole treatment.

Secondary endpoints included pathological outcome, surgical complications, and relation to high-risk MRI features.

## 3. Results

A total of 50 patients who received neoadjuvant chemoradiation and had available MRI images were identified. Based on the sarcopenia t-score, 12 (24%) patients were sarcopenic at staging (SG) and 16 (32%) on the restaging scan, while 38 patients (76%) represented the non-sarcopenic group (NSG). Patient characteristics are described in Table 1.

The majority of patients were male (60%) with an average age of 59.7 ± 10.4 years. Patients with initial sarcopenia had significantly lower body mass index (BMI) and smaller psoas muscle area than the NSG (*p* = 0.004 and *p* = 0.001, respectively). In addition, there were statistically significant differences regarding post-treatment sarcopenia between the two groups. None of the sarcopenic patients recovered on the post-treatment scan; more so, four patients became sarcopenic after nCRT (*p* < 0.001). No further statistically significant differences were noted among the remaining baseline characteristics.

Induction chemotherapy was administered in 20% of patients, consolidation in a third of patients, while 24% received chemotherapy both prior to chemoradiation and after (Table 2). There were no statistically significant differences between SG and NSG regarding the number of chemotherapy cycles (0.468). Forty-two percent of patients received more than four cycles of chemotherapy. All patients underwent long-course radiotherapy, receiving 50 Gy.

**Figure 2:**
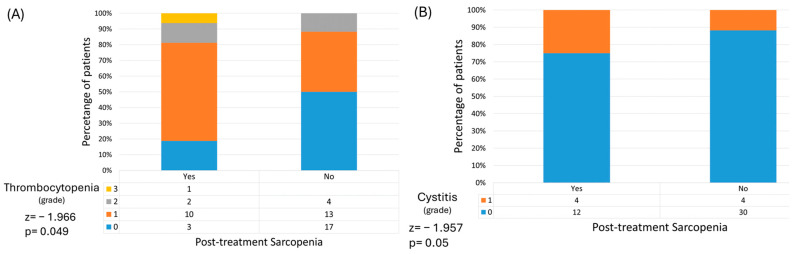
Post-treatment sarcopenia is associated with higher rates of thrombocytopenia (**A**) and cystitis (**B**).

Toxicity-wise, both SG and NSG had similar rates of acute adverse events; no statistically significant differences were observed. However, thrombocytopenia and cystitis were significantly associated with post-treatment sarcopenia (*p* = 0.049 and *p* = 0.05, respectively) (Figure 2). Moreover, while not statistically significant, G3 and above adverse events were more present in patients who became sarcopenic following radiotherapy, suggesting that those who lose psoas muscle mass over the course of treatment also experience more severe side effects (OR 2.5, *p* = 0.446).

Although the oCR was higher in the NSG compared to the SG, the difference between the two groups was not statistically significant (23% vs. 0%, *p* = 0.09) (Table 3).

Overall complete response rates were significantly correlated with post-treatment sarcopenia (OR 0.08, *p* = 0.043).

**Figure 3:**
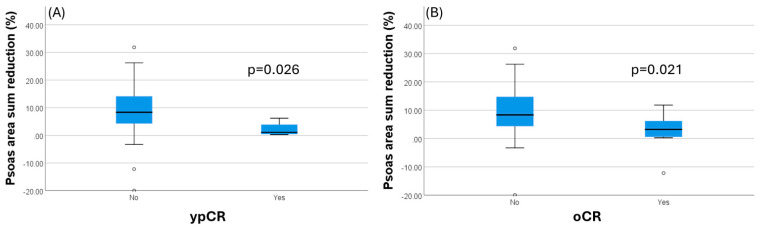
Percentage psoas muscle area reduction is significantly greater in patients not achieving a complete pathological response (**A**). The same applies when considering the overall complete response (cCR and pCR) (**B**).

Furthermore, there is a significant inverse association between the percentage of psoas muscle loss and pCR (*p* = 0.029) and oCR (*p* = 0.022), as seen in Figure 3. The higher the psoas muscle loss is, the lower the rates of pCR and oCR are. Patients who had a pCR had a mean loss of PMA of 2.1% (±5.5), while those who did not experience pCR had a loss of 8.7% (±17.8). Overall complete response was identified in patients having a PMA loss of 2.88% (±13.7) between pre- and post-neoadjuvant therapy, while a mean loss of 9.39% (±17.5) was observed in patients not achieving a complete response. Post-treatment response assessed by MRI tumor regression grade (mrTRG) showed no significant difference between SG and NSG; however, it is noted that the number of mrTRG scores of 0 and 1 was four times higher in the non-sarcopenic patients.

Out of the 50 initial patients, only 42 underwent surgery. Five had a complete clinical response, two had advanced, inoperable disease, and one declined surgery. Moreover, 57% of surgical patients underwent abdomino-perineal resection. There is no statistically significant difference with regard to post-operative morbidity, pathological T- or N-stage, CRM, EMVI status, resection margins, or Ryan regression grade between SG and NSG. There was a tendency for sarcopenic patients to have lower rates of tumor downstaging and negative CRM (OR = 0.194, *p* = 0.071 and OR = 16, *p* = 0.052), compared to NSG, which was not significant. Table 4 shows the surgical and pathological findings in detail.

## 4. Discussion

In our study, we found that the overall complete response rate is negatively associated with the presence of sarcopenia after neoadjuvant treatment and that an increase in psoas muscle loss during neoadjuvant treatment translates into lower rates of both pathological and overall complete responses. On average, an 8% decrease in PMA precluded a complete response. Even though there is no statistical significance, it is important to note that all patients with a complete response were non-sarcopenic at baseline.

Sarcopenia also influences toxicity; we have found significant associations between post-treatment sarcopenia and increased rates of cystitis and thrombocytopenia. This alludes to the cumulative effect that radiotherapy and chemotherapy have together with cancer-induced sarcopenia.

Our data align with previous literature published on the effect of sarcopenia on locally advanced rectal cancer treatment outcomes. To date, there are only three studies evaluating how sarcopenia impacts tumor response rates in locally advanced rectal cancer treated with chemotherapy, radiotherapy, and surgery.

The first to report the detrimental effect sarcopenia has on achieving a pathologic response was Olmez et al. [15]. In a retrospective study of 61 LARC patients, they discovered that the pCR rate was significantly higher in the non-sarcopenic group compared to the sarcopenic group, with rates of 21.4% versus 3.0% (*p* = 0.025). Subsequent research from Bedrikovetski et al. [16] failed to confirm these findings. Their retrospective analysis of 167 cases of LARC found no statistically significant difference in pathological good tumor regression grades (i.e., TRG0/1) between SG and NSG. Both studies collected data from patients that received only nCRT followed by surgery, without neoadjuvant chemotherapy. Since the introduction of the total neoadjuvant treatment approach, pCR and cCR have become increasingly attractive endpoints for treatment evaluation in LARC. Sarcopenia was also analyzed in the context of TNT by Bedrikovetski et al. [14] in a prospective observational study on 118 patients with LARC undertaking TNT. They found a significantly higher overall complete response rate in the non-sarcopenic group compared with the sarcopenic group (*p* = 0.001). Our study showed an association between oCR and post-treatment sarcopenia. This is likely due to the small cohort size, considering that post-neoadjuvant therapy, the number of sarcopenic patients grew by 4, to a total of 16. The association between psoas muscle loss during neoadjuvant treatment and poorer oncological outcomes has been investigated in a study by Yassaie et al. [19], showing that the average PMA loss was 5% and that patients with complete responses had significantly less muscle loss (2.8%) than patients with persisting tumors (6.8%), *p* = 0.02. Our results are in accordance with these findings, as we observed that patients with pCR had a mean loss of PMA of 2.1%, while those with residual tumors had a loss of 8.7%, *p* = 0.029.

Pathological complete response has been shown to strongly correlate with disease-free survival (DFS) and overall survival (OS) [22,23]. However, it might not be the optimal surrogate for OS or the preferred outcome for every patient [24]. As evidence on the promising results of watch-and-wait strategies continues to grow, total neoadjuvant therapy, with the goal of achieving a complete clinical response and preserving the rectum, is increasingly becoming a valid option [25,26]. Nonetheless, the absence of a consensus on the definition of complete clinical response hampers consistency and the ability to compare outcomes across different trials [17,27].

Looking beyond pCR, cCR, and sarcopenia, Gartrell et al. [28] investigated in a retrospective study on 132 patients with LARC whether sarcopenia can predict a poorer overall survival. This analysis was positive, with sarcopenia being identified as an independent risk factor for worse overall survival, with a statistically significant hazard ratio (HR) of 3.71, *p* = 0.016. A meta-analysis on surgical LARC candidates demonstrated a significantly lower overall survival for patients with pre-operative sarcopenia (HR = 2.07, *p* < 0.001) [29].

Sarcopenia is usually defined on CT images, at the level of L3 vertebra, and several variables and indexes are calculated, such as psoas muscle area (PMA), psoas muscle index (PMI), total psoas muscle area (TPMA), total psoas muscle index (TPMI), skeletal muscle area (SMA), skeletal muscle index (SMI), and volumetry [9,14,16,19,28,30]. Sarcopenia assessment in our study differed by using MRI images instead, and measurements were performed at the L4 vertebra. MRI was chosen because of better soft tissue definition and availability of images performed mainly at the 8-week post-nCRT timepoint. We opted for the level of the L4 vertebra considering the increased proximity to the radiation fields to better capture the effect of radiotherapy on sarcopenia. As per the European Working Group on Sarcopenia in Older People (EWGSOP) 2 guidelines [7], CT and MRI are validated, the gold standard for noninvasive assessment of muscle quantity/mass. While the L3 vertebra is usually the reference level, and the latest EWGSOP2 guidelines recommend DXA to assess muscle quantity, several authors have used the psoas muscle area or index at L3 or L4 to reliably define sarcopenia and correlate it with poor treatment outcomes in rectal cancer [14,19].

While there is strong evidence that sarcopenia increases chemotherapy-induced toxicity in colorectal cancer [31], the effect of radiotherapy is less conclusive. Our data are in accordance with Bedrikovetski et al. [14] and Sandoval et al. [32], who showed that there is no significant association between sarcopenia and grade  ≥  3 radiation-induced toxicity. In our cohort, however, increased rates of cystitis and thrombocytopenia were associated with post-treatment sarcopenia.

There is great variability in the age of the cohort, ranging from 50 to 70 years. Sarcopenia is known to be more prevalent as age increases; hence, the presence of sarcopenia could be caused by the increased age [33]. There were, however, no significant differences in age between the non-sarcopenic and sarcopenic groups.

Sarcopenic patients had also noticed more advanced disease, which in turn could have prevented them from achieving a complete response, as it is known that a higher T- and N-stage are associated with poorer overall response rates [34,35]. The higher rates of sarcopenia could be a result of more aggressive disease and a higher tumor burden.

The BMI also significantly varied between the groups. Non-sarcopenic patients had an average BMI of 28.1 kg/m^2^, while the average BMI in the sarcopenia group was 24.9 hg/m^2^. While these values are in the normal and overweight areas, they do suggest a poorer nutritional status in the sarcopenic group. The impact of BMI on the oncologic outcome of rectal cancer patients remains disputed, with some studies showing higher rates of locoregional recurrence while others show better DFS and OS [36,37].

Our study is not without limitations. Firstly, although the data came from a single-institution database, retrospective data collection has its inherent pitfalls. Secondly, because of the highly specific patient population, our cohort was small and corresponded to a low proportion of oCR. Sample size is underpowered due to lack of patients with complete medical records (limited number of patients with evaluable pre- and post-treatment MRI scans). Therefore, the small sample size reduces generalizability of the data, and the results need to be interpreted with caution. Thirdly, we performed the assessment of sarcopenia using only one variable. Had there been more variables/indexes assessed, it might have led to a better characterization of the cohort. Prospective studies are required to confirm these findings.

## 5. Conclusions

Post-treatment sarcopenia and psoas muscle loss during neoadjuvant therapy were negatively associated with tumor response in locally advanced rectal cancer. Thrombocytopenia and cystitis are more frequent in post-treatment sarcopenic than non-sarcopenic patients undergoing neoadjuvant chemoradiation for rectal cancer.

## Figures and Tables

**Table 1:** Baseline characteristics.

Variables	Total (n = 50)	Sarcopenic Group (n = 12)	Non-Sarcopenic Group (n = 38)	*p*-Value
Age (years), mean (SD)	59.7 (10.4)	59.3 (9.9)	59.8 (10.7)	0.785
Gender				0.74
Male	30 (60%)	8	22	
Female	20 (40%)	4	16	
ECOG				0.999
0–1	45	8	37	
2	5	0	5	
BMI, (kg/m^2^)	27.4 (17.9–39.1)	24.9 (18.1–32.1)	28.1 (17.9–39.1)	**0.04**
Psoas muscle area (cm^2^)	23.7 (13–40.8)	18 (13–22)	25.5 (15.3–40.8)	**0.001**
Post-treatment sarcopenia				**<0.001**
Yes	16 (32%)	12 (100%)	4 (10.5%)	
No	34 (68%)	0 (0%)	34 (89.5%)	
Clinical T stage				0.163
T2	8 (16%)	2 (16.7%)	6 (15.8%)	
T3	29 (58%)	5 (41.7%)	24 (63.2%)	
T4a	6 (12%)	1 (8.3%)	5 (13.2%)	
T4b	7 (14%)	4 (33.3%)	3 (7.9%)	
Clinical N stage				0.311
N0	19 (38%)	0 (0%)	8 (21.1%)	
N1	6 (12%)	5 (41.7%)	12 (31.6%)	
N2a	17 (34%)	1 (8.3%)	5 (13.2%)	
N2b	8 (16%)	6 (50%)	13 (34.2%)	
AJCC 8th ed.				0.413
I	2 (4%)	0	2 (5.3%)	
II	6 (12%)	0	6 (15.7%)	
III	42 (84%)	12 (100%)	30 (79%)	
Tumor location				0.808
Lower rectum	27 (54%)	7 (58.4%)	20 (51.5%)	
Middle rectum	16 (32%)	4 (33.3%)	12 (31.6%)	
Upper rectum	7 (14%)	1 (8.3%)	6 (15.8%)	
MRF				0.496
MRF positive	19 (38%)	6 (50%)	13 (34.2%)	
MRF negative	31 (62%)	6 (50%)	25 (65.8%)	
EMVI				0.718
EMVI positive	14 (28%)	4 (33.3%)	10 (26.3%)	
EMVI negative	36 (72%)	8 (66.7%)	28 (73.7%)	
TD				0.999
TD positive	3 (6%)	0 (0%)	3 (8%)	
TD negative	47 (94%)	12 (100%)	35 (92%)	
Cranio-caudal tumor extension (mm)	61.2 (23–140)	70.6 (35–140)	58.2 (23–115)	0.275

Note: Values are given as n (%) or mean (SD). Bold *p*-values represent statistically significant results. Abbreviations: AJCC, American Joint Committee on Cancer; BMI, body mass index; ECOG, Eastern Cooperative Oncology Group performance status; MRF, mesorectal fascia; EMVI, extra-mural venous invasion; TD, tumor deposit.

**Table 2:** Treatment compliance and toxicity.

Variables	Total (n = 50)	Sarcopenic Group (n = 12)	Non-Sarcopenic Group (n = 38)	*p*-Value
Neoadjuvant chemotherapy				0.598
Induction	10 (20%)	2 (16.7%)	8 (21.1%)	
Consolidation	18 (36%)	5 (41.7%)	13 (34.2%)	
Both	12 (24%)	4 (33.3%)	8 (21.1%)	
None	10 (20%)	1 (8.3%)	9 (23.7%)	
Compliance to chemotherapy				0.468
≤4 cycles	29 (58%)	2 (4%)	12 (24%)	
>4 cycles	21 (42%)	10 (20%)	26 (52%)	
Compliance to radiotherapy				
50 Gy	50 (100%)			
Duration of radiotherapy (days)	38 (9–86)	34.6 (9–45)	39 (31–86)	0.664
Timing of surgery starting from the end of chemoradiation (weeks)	17.1 (6–54)	22.5 (6.4–54)	15.2 (6–40.1)	0.451
Anemia (worst grade)				0.627
0	8 (16%)	1 (8.3%)	7 (18.4%)	
1	28 (56%)	6 (50%)	22 (57.9%)	
2	11 (22%)	4 (33.4%)	7 (18.4%)	
3	3 (6%)	1 (8.3%)	2 (5.3%)	
Neutropenia (worst grade)				0.388
0	38 (76%)	10 (83.3%)	28 (73.7%)	
1	6 (12%)	0	6 (15.8%)	
2	5 (10%)	2 (16.7%)	3 (7.9%)	
3	1 (2%)	0	1 (2.6%)	
Thrombocytopenia (worst grade)				0.222
0	20 (40%)	3 (25%)	17 (44.7%)	
1	23 (46%)	6 (50%)	17 (44.7%)	
2	6 (12%)	2 (16.7%)	4 (10.6%)	
3	1 (2%)	1 (8.3%)	0	
Neuropathy (worst grade)				0.463
0	31 (62%)	6 (50%)	25 (65.8%)	
1	18 (36%)	6 (50%)	12 (31.6%)	
2	1 (2%)	0	1 (2.6%)	
Diarrhea (worst grade)				0.795
0	46 (92%)	11 (91.7%)	35 (92.1%)	
1	3 (6%)	1 (8.3%)	2 (5.3%)	
2	1 (2%)	0	1 (2.6%)	
Nausea and vomiting (worst grade)				0.851
0	45 (90%)	11 (91.7%)	34 (89.5%)	
1	4 (8%)	1 (8.3%)	3 (7.9%)	
2	1 (2%)	0	1 (2.6%)	
Proctitis (worst grade)				0.593
0	28 (56%)	6 (50%)	22 (57.9%)	
1	17 (34%)	4 (33.3%)	13 (34.2%)	
2	4 (8%)	2 (16.7%)	2 (5.3%)	
3	1 (2%)	0	1 (2.6%)	
Cystitis (worst grade)				0.999
0	42 (84%)	10 (83.3%)	32 (84.2%)	
1	8 (16%)	2 (16.7%)	6 (15.8%)	

Note: Values are given as n (%) or mean (SD).

**Table 3:** Treatment response.

Variables	Total (n = 50)	Sarcopenic Group (n = 12)	Non-Sarcopenic Group (n = 38)	*p*-Value
oCR				0.092
Yes	9 (18%)	0	9 (24%)	
No	41 (82%)	12 (100%)	29 (76%)	
cCR	4 (8%)	0	4 (10%)	0.319
pCR	5 (10%)	0	5 (13%)	0.560

Note: Values are given as n (%) or mean (SD). Abbreviations: oCR, overall complete response; cCR, clinical complete response; pCR, pathological complete response.

**Table 4:** Surgical and pathological outcomes.

Variables	Total (n = 50)	Sarcopenic Group (n = 12)	Non-Sarcopenic Group (n = 38)	*p*-Value
Procedure				0.825
Restorative	18 (36%)	3 (25%)	15 (39.5%)	
Non-restorative	24 (48%)	7 (58.3%)	17 (44.7%)	
No surgery	8 (16%)	2 (16.7%)	6 (15.8%)	
Post-operative morbidity ^a^				0.657
Yes	12 (38%)	6 (60%)	24 (75%)	
No	30 (62%)	4 (40%)	8 (25%)	
ypT stage ^a^				0.100
T0	6 (14.3%)	0 (0%)	6 (18.8%)	
T1	1 (2.4%)	1 (10%)	0 (0%)	
T2	12 (28.6%)	2 (20%)	10 (31.2%)	
T3	18 (42.9%)	4 (40%)	14 (43.8%)	
T4a	2 (4.8%)	1 (10%)	1 (3.1%)	
T4b	3 (7.1%)	2 (20%)	1 (3.1%)	
ypN stage ^a^				0.460
N0	29 (69%)	8 (80%)	21 (65.6%)	
N1	7 (16.7%)	1 (10%)	6 (18.8%)	
N2a	4 (9.5%)	0	4 12.5%)	
N2b	2 (4.8%)	1 (10%)	1 (3.1%)	
CRM ^a^				0.052
positive	2 (4.8%)	2 (20%)	0 (0%)	
negative	40 (95.2%)	8 (80%)	32 (100%)	
ypEMVI ^a^				0.238
positive	1 (2.4%)	1 (10%)	0 (0%)	
negative	41 (97.6%)	9 (90%)	32 (100%)	
Resection margins ^a^				0.540
R0	39 (92.8%)	12 (100%)	27 (92%)	
R1	3 (7.2%)	0	3 (10%)	
Modified Ryan regression grade ^a^				0.193
0-1	12 (28.5%)	1 (10%)	11 (34.4%)	
2	16 (38%)	6 (60%)	10 (31.2%)	
3	14 (33.5%)	3 (30%)	11 (34.4%)	

Note: Values are given as n (%) or mean (SD). Restorative procedures: anterior resections, rectosigmoid resections. Non-restorative procedures: abdominoperineal resections, proctocolectomies (one case). ^a^ Only surgical patients (n = 42).

## Data Availability

The data presented in this study are available upon request from the corresponding author. The data are not publicly available due to patient privacy.
